# Acid-Fast Bacilli in Non-Tuberculous Sputum

**Published:** 1909-07-03

**Authors:** 


### ACID-FAST BACILLI IN NON-TUBERCULOUS SPUTUM.

An acid-fast bacillus is one which retains the stain in spite of treatment with strong sulphuric acid, as in the well-known Ziehl-Neelsen method of staining tubercle bacilli. The best-known acid-fast bacillus is undoubtedly the tubercle bacillus; but there are other bacteria that retain this stain. The bacillus of leprosy is one of these, and the smegma bacillus is another; a bacillus that is sometimes found in cow's milk or in butter is a third. Indeed, there are so many acid-fast bacilli that a book has been written upon them alone.

The difficulties that may arise in distinguishing between tubercle bacilli and smegma bacilli in the urine are familiar. It is less well known, perhaps, that it is by no means every acid-fast bacillus in sputum that is a tubercle bacillus. It is quite true that the very great majority of sputa containing acid-fast bacilli come from phthisical persons. It is equally true that it is the general rule to assume that the discovery of acid-fast bacilli in sputum is certain proof of phthisis in the individual from whom the sputum came. The possibility of falling into error by too slavishly adhering to this rule, however, should be borne in mind, for there are exceptions, and the significance of these, in connection with life insurance for instance, is very great. There may even be obviously abnormal lung signs in association with acid-fast bacilli in the sputum, and yet the pulmonary lesion need not be tuberculous.

The following case would serve admirably for a discussion of this point. A man aged 55 is recorded by Dr. Basile, in Naples, as having suffered from bronchitis in former years, and he now exhibited clinical signs of granular kidney and glycosuria. His condition improved for some months as the result of careful dieting, but then, to the alarm of all, he developed a much more serious attack of bronchitis than he had had before, with abundant expectoration and some pyrexia. Acid-fast bacilli were found in the sputa, and it seemed clear that the patient had developed pulmonary tuberculosis. The physical signs supported this diagnosis.

The sputa soon became fœtid, and upon analysis they were found to contain lung tissue. The prognosis naturally seemed to be of the gravest, but the lesion localised itself completely, and ultimately cleared up, the patient convalescing gradually and recovering in due course.

It might be urged at once that this was nothing more than one of those cases of surprising recovery from an apparently hopeless degree of pulmonary tuberculosis, and that the acid-fast bacilli were really tubercle bacilli after all. Extensive laboratory investigations were carried out upon the organism, however, and there seems to be no doubt that culturally and biologically the bacilli were some other acid-fast variety. Not only were they decolorised by 2 per cent. lactic acid in alcohol in a few minutes, whereas tubercle bacilli are not similarly decolorised in less than half an hour, but also they grew with far greater ease than do tubercle bacilli as a rule. In broth and on gelatine and on glycerine-agar they gave cultures in four and twenty hours, whilst when they were injected into guinea-pigs they caused no other pathogenic effect than a feeble and local lesion. Tubercle bacilli cannot be cultivated very easily, and the cultures do not grow fast; and ordinary tubercle bacilli are very pathogenic for guinea-pigs, giving rise not merely to a local lesion, but to a general tuberculosis.

